# Small scale water treatment practice and associated factors at Burie Zuria Woreda Rural Households, Northwest Ethiopia, 2015: cross sectional study

**DOI:** 10.1186/s12889-016-3571-2

**Published:** 2016-08-26

**Authors:** Hailegebriel Belay, Zewdu Dagnew, Nurilign Abebe

**Affiliations:** 1 Burie Woreda, Burie, Ethiopia; 2 Public health department, Medicine and Health Sciences College, Debre Markos University, Debre Markos, Ethiopia

**Keywords:** Small scale, Water treatment, Rural, Factors, Burie

## Abstract

**Background:**

Consuming unsafe water results in infections that lead to illness or death from water borne diseases. Though there is an increasing effort from Ethiopian government to access safe water still there are households with limited access of safe water as a result, they depend on rain, well and spring water source for domestic use. However, the water treatment practice with the available technology is not studied before in the study area. This study was conducted in rural area where there was no improved water source for domestic consumption. Households’ access water from rain, spring, river and well water which need some ways of action to make water safe for the intended utilization termed as treatment. Hence, the aim of this study was to assess magnitude of small scale water treatment practices and associated factors at household level in Burie zuria woreda, North West Ethiopia, 2015.

**Methods:**

Community based cross-sectional study design with multi-stage sampling technique was used to evaluate water treatment practice and associated factors among rural households in Burie Zuria Woreda. A total of 797 households included in the study. Completeness of questionnaires were checked daily and data were coded and entered into Epi-Data and transported to SPSS version 16 software package for further analysis. Binary and multivariable logistic regression models fit to identify associated factors at 95 % CI and *P*-value <0.05.

**Result:**

A total of 797 out of 846 participants responded to a questionnaire with a response rate of 94.2 %. The mean age of respondents was 44.9(SD ±10.7) years. Among the total study participants, 357(44.8 %) of them were practicing small scale water treatment at household level. Methods of water treatment at household level were; chlorine, boiling and let stand and settle. Associated factors were female headed households practice water treatment than male headed households (AOR = 1.80, 95 % CI = 1.24–2.62), educational status of being literate was associated with water treatment than illiterates (AOR = 2.07, 95 % CI = 1.51–2.83), dipping of water was associated with water treatment practice than pouring from the water collection jar (AOR = 4.11, 95 % CI = 2.89–5.85) and those households more frequently fetch water were practicing water treatment than those fetch less frequently (AOR = 4.90, 95 % CI = 2.92–8.22) and (AOR = 3.76, 95 % CI = 1.97–7.18) respectively were found to be significantly associated with small scale water treatment practice at household level.

**Conclusions:**

Small scale water treatment at household level is still low in the study area. Females headed households, educated people, dipping from the jar and those who fetch water more than twice a day were significant factors for water treatment. Therefore females’ practice should be maintained and scale up for male headed households. Those with no primary education need special emphasis to educate them on the importance of water treatment. Encourage education through non formal mechanisms for rural people are also recommended.

## Background

Treating water and safely storing it in the home are commonly referred to as “household water treatment and safe storage” (HWTS) or treating water at the “point of use”. Although HWTS is not new, its recognition as a key strategy for improving public health is just emerging. For centuries, households have used a variety of methods for improving the appearance and taste of drinking water. Even before germ theory was well established, successive generations were taught to boil water, expose it to the sun or store it in metal containers with biocide properties, all in an effort to make it safer to drink [1].

Treat water that has become contaminated both at the source as well as through domestic handling with the goal of reducing contamination to levels of low microbial risk is said to be household water treatment [2]. Human being have a right to water and entitles everyone to have sufficient, safe, acceptable, physically accessible and affordable water for personal and domestic uses [3, 4].

Lack of clean drinking water, poor sanitation facilities and lack of community education programs are contributing to continued outbreaks of acute watery diarrhea in some parts of Ethiopia [5]. Water supply and sanitation situation in Ethiopia is very poor, as most of the population does not have access to safe and adequate water supply and sanitation facilities. As a result three-fourth of the health problems in Ethiopia is due to communicable diseases attributable to unsafe or inadequate water supply and improper waste management particularly excreta [6].

Ethiopian rural water coverage has increased at promising rates since 1990, from 8 to 26 % according to joint monitoring program (JMP/UNICEF-WHO) figures, and from 11 to 62 % according to government figures [7]. Even though there is great discrepancy between the two reports; both figures indicate that there is problem in water coverage in rural Ethiopia. To have access to safe drinking water does not only imply microbial and chemically safe water, but also to have a secured supply and public access to the water sources. Household treatment of water is widespread over the world, but in Ethiopia, only 5 % of the population make use of it. Nevertheless, access to safe drinking water is very low [8].

This study is first in its kind as far as the researchers’ knowledge of searching in the scientific published papers there was no published article in this area. Since the study was done in the rural area with sole source of water is not piped which is river, spring, rain water and hand dung well source of drinking water, that needs treatment at point of use. it can be used as initial point to change the peoples’ behavior to domestic water treatment. However, the practice of making water “safe” to dink-treatment is not studied in the study area. The government encourages, domestic water treatment using chlorine chemicals, boiling, and other means and proper utilization water through the rural health extension program. Therefore, recommended water treatment technologies in the package health extension manual are; boiling, expose for sun light, use of chlorine, filtration and other activities the society believe that make drinking water safe. Therefore, this stud tried to identify how the rural people use the available water treatment technology and factors associated with small scale/household level water treatment practice.

## Methods

### Study design

Community based quantitative cross- sectional study design was employed to determine the magnitude of small scale water treatment practice and associated factors.

### Study area and period

The study was conducted in Burie Zuria woreda (Woreda is second smallest administrative structure of Ethiopian government system next to Kebele). It is about 407 kilometers away from Addis Ababa-the capital city of Ethiopia in the North-Western direction and the study period was January 1 to March 15, 2015. The woreda has one urban and 18 rural Kebele (Kebele is the smallest administrative structure in Ethiopian government system) and the communities engage in animal rearing and crop production activities the total population of the woreda is 139,235. The woreda Water coverage (availability with 30 min travel or 1.5 Km away from households) and access (able to reach and use the available water) is 80 % and 84 % respectively as the information obtained from Woreda water office. According to 2007 national censes, Burie woreda has a total population of 143,132 of whom 71,208 are men and 71,924 are women; 25,975 (18.15 %) are urban inhabitants. Source of water is river, spring, hand dung well and rain water. The scarcity is there also especially at times of dry season (December to June) the study was done during these period.

### Populations

Source populations were all rural households in Burie zuria Woreda and Study population was households who were living in the selected kebeles that fulfilled the inclusion criteria. Sampling unit was households and study units were head of the household. Households resided more than 6 months and age above18 years old were include since they can give complete image of the community and ethically accepted to participate in studies respectively. Head of households were interviewed with the assumption of getting correct response from them. Priority was given for women because they are more familiar with water handling than men. However where there is household with no women for any reason men were considered for interview. And individuals who were not accessed after second visit and those unable to communicate were excluded in the study.

### Sample size and sampling technique

#### Sample size

The sample size was calculated using single population proportion formula [9] by considering the following assumptions.

$$ \mathrm{n}=\frac{\left({\mathrm{Z}}^2\right)\mathrm{xpxq}}{{\mathrm{d}}^2} $$

$\frac{\left(1{.96}^2\right)\mathrm{x}50\%\mathrm{x}50\%=384}{{\left(5\%\right)}^2}$, considering design effect 2 and 10 % of non-response rate, =846 households.

Since there is no previous similar study, 50 % was taken as proportion (p), P = proportion of water treatment practice at household level, n = the required sample size, Z = A standard score corresponding to 95 % confidence level and d = margin error of 5 %.

#### Sampling method and procedure

Multi stage sampling technique was applied followed by systematic random sampling techniques. There were 19 kebeles in the woreda and out of these 20 % of the kebeles which are four in number (winma-Abay, Alefa, Gulem and Shakua) were selected by simple random sampling techniques and the household were selected by systematic random sampling method to get the desired study population. There were 1886 households in Winma-Abay kebele from this 272 households were selected. 1200 households were from Alefa kebele and 173 households were selected. 1120 and 1560 households were in shakua and Gulem kebeles respectively then 166 and 230 households were taken that gives 846 total study households.

### Variables

#### Dependent variable

Small scale water treatment practice (Yes/No).

#### Independent variables

Socio-demographic (Age, sex, educational status, and religion, marital status, occupational status, family size, head of a household and monthly income) and Environmental issues: Source of drinking water, days when water is not fetch, time taken for fetching, and ways of fetching the water from the collection jar.

### Operational definition

Small scale water treatment was dictated as “Yes” for the question “Do you do anything to your water to make it safer to drink” if at least one of the following options was practiced at household level during data collection time. These were boiling, bleach/chlorine (Bishagary31 and Wuhager29), solar disinfection, stand and settle and strain through cloth filter. Options of water treatment were taken from WHO tool kit for household water treatment and storage evaluation program [10].

### Data collection and measurement

Nine grade ten completed data collectors and two diploma holders nurse supervisors were involved for data collection process. The training was given for two days for all data collectors and supervisors. The questionnaire was prepared based on the available literature reviewed to elicit magnitude and associated factors of water treatment. A conceptual framework was used for the development of the questionnaire. Data collection was done by pre tested and pre coded interview administered questionnaire. The questionnaire was originally prepared in English language and then translated to the local language (Amharic) and again translated to English for consistency.

### Data quality assurance

The quality of data were assured by proper designing and pre-testing of the questionnaires and by giving training for the data collectors and supervisors before the actual data collection. Every day after data collection, questionnaires were reviewed and checked for completeness and relevance by the supervisors and principal investigator and the necessary feedback was offered to data collectors in the next morning. Also, the principal investigators and an experienced data clerk were carefully entered and thoroughly cleaned the data before the commencement of the analysis.

### Data processing and analysis

Completeness of questionnaires was checked visually and data were coded and entered into Epi-data version 3.5.4 and transported to SPSS version 16 software package for analysis. For controlling errors 10 % of the questionnaire was double entered, also frequency checks were done. Variables with *P*-value <0.25 in bivariate analysis were entered to multivariate analysis and *p*-value of 0.05 at 95 % CI and odds ratio were used to declare statistically significance.

### Ethical consideration

The proposal was approved by Ethical Review Committee of College of Medicine and Health Science, Debre Markos University. All the study participants were informed about the purpose of the study and finally their oral consent was obtained before collecting data. The respondents informed that they have the right to refuse or terminate at any point of the data collecting. The information provided by each respondent was kept confidential. The dissemination of the finding does not also refer specific respondent.

## Result

### Socio-demographic characteristics respondents

A total of 797 participants responded to a questionnaire with a response rate of 94.2 %. The mean age of respondents was 44.9 (SD, ±10.7) years. Among the total respondents, 597(74.9 %) of them were male and majority 652 (81.8 %) of them were married. Some 281(35.3 %) of them were illiterate. By occupational status of the respondents, 737(92.5 %), 31(3.9 %) and 29(3.6 %) were farmers, daily laborers and merchants respectively. Most 531(66.6 %), of them had a family size of ≥5 and 647(81.2 %) a head of household father and 406(50.9 %) of the respondents monthly average income was ≥1000 ETB (Table 1).

**Table 1:** Socio-demographic characteristics of respondents on small scale water treatment practice and associated factor at house hold level at Burie zuria woreda rural HHs, west Gojjam zone, Northwest Ethiopia, 2015 (*N* = 797)

Variable	Category	Frequency	%
Sex	Male	597	74.9
	Female	200	25.1
Age	18–30 years	75	9.4
	31–45 years	348	43.7
	46–65 years	355	44.5
	>65 years	19	2.4
Educational status	Illiterate	281	35.3
	Literate	516	64.7
Religion	Orthodox	791	99.2
	Muslim	6	0.8
Marital status	Married	652	81.8
	Single	54	6.8
	Divorced	54	6.8
	Widowed	37	4.6
Occupational status	Farmer	737	92.5
	Daily labourer	31	3.9
	Merchant	29	3.6
Number of individuals in the family	1	31	3.9
	2–4	235	29.5
	≥5	531	66.6
Head of a household	Father	647	81.2
	Mother	150	18.8
Monthly income	≤500 ETB	4	0.5
	501–999 ETB	387	48.6
	≥1000 ETB	406	50.9

### Environmental characteristics

Among the total study participants, majority 716(89.8 %) of them were getting water from hand dug well and 578(72.5 %) reported that there were days in week households did not fetch water for religious purpose which were mostly (56.7 %) was on weekend (Table 2) and some monthly holidays.

**Table 2:** Environmental characteristics of respondents on small scale water treatment practice and associated factor at house hold level at Burie zuria woreda rural Northwest Ethiopia, 2015

Variables	Category	Frequency	%
Source of drinking water	Protected well	716	89.8
	Unprotected well/spring	5	0.6
	Rain water	76	9.6
Are there days of the week water source having no services?	No	219	27.5
	Yes	578	72.5
Which days source of water is off (*N* = 578)	Saturday and Sunday	501	86.7
	Holly day and Sunday	77	13.3
Experience of storing water for three days	No	307	38.5
	Yes	490	61.5
Type of container for storing the water (*N* = 490)	Clay pot	264	53.9
	Iron container	18	3.7
	Jerri can	208	42.4
Number of containers (*N* = 490)	One	99	20.2
	Two	339	69.2
	Three	52	10.6
Capacity of the container (*N* = 490)	≤25 Liters	200	40.8
	>25 Liters	290	59.2
Containers covered (*N* = 490)	No	5	1
	Yes	485	99
Days the water is stored (*N* = 490)	For two days	36	7.3
	For three days	358	73.1
	More than three days	96	19.6
Washing status of the container (*N* = 490)	No	1	0.2
	Yes	489	99.8
Materials used for washing the container	Vegetation	427	87.1
	Chemicals	4	0.8
	only water	47	9.6
	with soap	12	2.4
Time taken to fetch the water	<30 minutes	773	97
	30–60 minutes	24	3
The type of container for fetching the water	Clay pot	165	20.7
	Jerri can	632	80.1
How many times do you fetch per day	One	181	22.7
	Two	395	49.6
	Three	151	18.9
	Four	70	8.8
The way of water dipping	Pouring	241	30.2
	Dipping	556	69.8
Design of water drawing material	With handle	685	85.9
	Without handle	112	14.1
Place for putting water drawing material	On shelf/table	728	91.3
	On the floor	63	7.9
	Inside the container	6	0.8

About, 490(61.5 %) of respondents had water storing experience out of which 264(53.9 %) stored in a clay pot. More than half 339(69.2 %), reported that they stored in two containers from which 290(59.2 %) of the containers had a capacity of storing ≥25 liters and from the observation during data collection period 485(60.85 %) water containers confirmed they had cover. The respondents water consumption per person per day was <10 liters. About 358(73.1 %) of households stored drinking water for three days and 489(99.8 %) responded that they washed the container before fetching water (Table 2).

### Small scale water treatment practice at household level

Among the total study participants, 357(44.8 %) of them treated water at their home. They use different modality of treatment approaches. More than half 213(59.7 %) boil water, 74(20.7 %) settle and stand and 70(19.6 %) have used chlorine chemicals (wuhaAger29 and Bishagary31) which are available in the local market for water treatment purpose. It was composite of Bishagary31 (44.3 %), Wuhager29 (41.4 %) and chlorine10 (14.3 %) from chemicals.

### Factors associated with small scale water treatment practice at household level

#### Bivariate analysis

Small scale water treatment practice at household level varied under the influence of various factors. In this test each independent variables were tested against the dependent variable. Accordingly sex, educational status, occupational status and the way of fetching, timing for fetching were found to have *P*-value <0.25 in which this variables were taken to multivariate analysis (Table 3).

**Table 3:** Bivariate Logistic Regression results on factors associated with small scale water treatment practice and associated factor at house hold level at Burie zuria woreda rural HHs, west Gojjam zone, Northwest Ethiopia, 2015 (*N* = 797)

Variable	Category	Water treatment status			COR (95 % CI)	*P*-value
		Yes	No	Total		
Sex	Male	289	308	597	1	
	Female	68	132	200	1.82(1.30–2.54)	0.000
Educational status of a respondent	Illiterate	219	191	410	1	
	Literate	138	249	387	2.06(1.55–2.75)	0.000
Occupational status of a respondent	Farmer	342	395	737	1	
	Daily laborer	6	25	31	3.60(1.46–8.89)	0.005
	Merchant	9	20	29	1.92(0.86–4.28)	0.109
The way of fetching the water	Pouring	158	83	241	1	
	Dipping	199	357	556	3.41(2.48–4.69)	0.000
Timing for fetching the water	Once a day	84	67	151	1	
	Twice a day	215	180	395	1.05(0.72–1.53)	0.801
	Three times a day	37	144	181	4.87(3.00–7.91)	0.000
	Four times a day	21	49	70	2.92(1.60–5.35)	0.000

#### Multivariate analysis

The method used was backward stepwise in order to get maximum significant variables. The results of multivariate model indicated that female headed households practice water treatment 1.24 times more likely than male headed households (AOR = 1.80, 95 % CI = 1.24–2.62), educational status of being literate were more than double to practice small scale water treatment at household level than those illiterate head of households (AOR = 2.07, 95 % CI = 1.51–2.83), dipping fetching water was associated with good practice of water treatment than pouring (AOR = 4.11, 95 % CI = 2.89–5.85) and frequency of fetching water more than three time a day was also positively associated with water treatment compared to less frequent (<3 times) a day (AOR = 4.90, 95 % CI = 2.92–8.22) furthermore, those fetch water four or more times a days were also prating water treatment almost 4 times than those fetch only once (AOR = 3.76, 95 % CI = 1.97–7.18) were found to be significantly associated with small scale water treatment practice at household level with *P*-value <0.05 (Table 4).

**Table 4:** Bivariate and Multivariable Logistic Regression results on factors associated with small scale water treatment practice and associated factor at house hold level at Burie zuria woreda rural HHs, west Gojjam zone, Northwest Ethiopia, 2015 (*N* = 797)

Variable	Category	Water treatment status		COR (95 % CI)	AOR (95 % CI)	*P*-value
		Yes	No			
Sex	Male	289	308	1	1	
	Female	68	132	1.82(1.30–2.54)	1.80(1.24–2.62)	0.002
Educational status of a respondent	Illiterate	219	191	1	1	
	Literate	138	249	2.06(1.55–2.75)	2.07(1.51–2.83)	.000
Occupational status of a respondent	Farmer	342	395	1	1	
	Daily laborer	6	25	3.60(1.46–8.89)	2.06(0.72–5.58)	0.183
	Merchant	9	20	1.92(0.86–4.28)	1.25(0.49–3.15)	0.630
The way of fetching the water	Pouring	158	83	1	1	
	Dipping	199	357	3.41(2.48–4.69)	4.11(2.89–5.85)	.000
Timing for fetching the water	Once a day	84	67	1	1	
	Twice a day	215	180	1.05(0.72–1.53)	1.17 (0.78–1.74)	0.438
	Three times a day	37	144	4.87(3.00–7.91)	4.90 (2.92–8.22)	0.000
	Four times a day	21	49	2.92(1.60–5.35)	3.76 (1.97–7.18)	.000

The result is interpreted as female respondents were 1.8 times more likely to practice small scale water treatment at household level compared to their counterparts males (AOR = 1.80, 95 % CI = 1.24–2.62) and literate respondents were 2.07 times more likely to practice small scale water treatment at household level compared to those who were illiterate (AOR = 2.07, 95 % CI = 1.51–2.83). Similarly, respondents who draw their water by dipping their container were 4.11 times more likely to practice small scale water treatment at household level compared to those who fetched their water by pouring their container (AOR = 4.11, 95 % CI = 2.89–5.85) and those respondents who fetched their water three times a day (AOR = 4.90, 95 % CI = 2.92–8.22) and four times a day (AOR = 3.76, 95 % CI = 1.97–7.18) were 4.90 and 3.76 times more likely to practice small scale water treatment at household level compared to those who fetched their water once a day.

## Discussion

Among the total study participants, 357(44.8 %) of them practiced small scale water treatment at household level. This finding is lower than MDG strategy that was planned to reduce the proportion of people without sustainable access to safe drinking water and proper sanitation to half of its number by 2015 [11] but higher than the findings from WHO estimate of China, middle and low income countries 20 % and 33 % respectively [12], Zambia 35.2 % [13] and Ethiopia where, 80 % of the rural and 20 % of urban population have no access to safe water; which is the least among the continent [14]. The possible explanations for this finding being lower might be related with sample size, study design and study period but for that of the study being higher might be related with sample size, study design and differences in year of study.

In this study about 59.7 % of the households used boiling as treatment strategy. About 20.7 % used settle and stand and 19.6 % used chlorine as domestic water treatment options. Majority of them used boiling this might be due to the method is easy to practice, mostly the households have the required material to do so and there is water boiling practice for other purposes like for coffee, washing heavily soiled utensils. This was in line with the suggestion given by CDC [15]. On the other hand none of them use solar system for water treatment. It can be due to non-availability of the technology and the population is from the rural Ethiopia. Some used chlorine since it is costly and available only in the urban market where most of the residents visit market rarely. There is also evidence of discomfort from change in taste and odor following the utilization of chlorine [16] that might contribute for less utilization compared to boiling. Even though there is a practice of settle and stand for household water treatment, it smaller relative to boiling. This might be due to little amount of water in the house to settle and wait till settlement. Additionally there was lack of appropriate water storage material in the house.

Female respondents were 1.8 times more likely to practice small scale water treatment at household level compared to their counterparts (AOR = 1.80, 95 % CI = 1.24–2.62). This finding was in line with the finding by Maria Elena Figueroa and D. Lawrence Kincaid [17]. The possible justification for the finding might be due to the fact that in most communities fetching and storing water at household level are the responsibilities of females and among the cares given to the family providing water treated at home is one of the critical cares.

Literate respondents were 2.07 times more likely to practice small scale water treatment at household level compared to those who were illiterate (AOR = 2.07, 95 % CI = 1.51–2.83). This finding was in line with the finding by Maria Elena Figueroa and D. Lawrence Kincaid [17]. The possible explanation for this finding might be due to the fact that literates might read leaflets and brushers and may better understand health risks of drinking contaminated water.

Respondents who draw their water by dipping their container were 4.11 times more likely to practice small scale water treatment at household level compared to those who draw their water by pouring their container (AOR = 4.11, 95 % CI = 2.89–5.85). This may be due to the fact that they might think that dipping the container for fetching may be liable to contaminants and to avoid those contaminants, respondents may employ either of the methods for water treatment at household level.

Those respondents who fetched their water three times a day (AOR = 4.90, 95 % CI = 2.92–8.22) and four times a day (AOR = 3.76, 95 % CI = 1.97–7.18) were 4.90 and 3.76 times more likely to practice small scale water treatment at household level compared to those who fetched their water once a day. The possible reasons for this may be those who fetched the water most frequently may have a chance to store their water which in turn enables them to treat their water by storing.

### Limitations of the study

- ✓ Cross-sectional nature of the study design could not show cause effect relationship

## Conclusion

Small scale water treatment at household level is still low. Female respondents were better practicing in small scale water treatment at household level than males. Educational status was also factor for water treatment practice in which, literate were better practicing small scale water treatment at household level than those who were illiterate. Who had an experience of drawing water by dipping were better practicing small scale water treatment at household level better than those who draw by pouring and those who were fetching the water more than two times a day were better practicing small scale water treatment at household level than those who were fetching once a day.

## Recommendation

- ➢ According to the results of this research,
  Woreda Health office of Burie Zuria Woreda should:-
  - ✓ Give attention for those who were illiterate about small scale water treatment at household level
  - ✓ Scale up experience of small scale water treatment at household level by females and literates to their counter parts
- ➢ Governmental and non-governmental organizations should:-
  - ✓ Advocate small scale water treatment practice at household level for males, illiterates and for those who fetched their water by pouring and once a day
- ➢ Households who were not practicing small scale water treatment at household level should:-
  - ✓ Treat water at their house before consumption
  - ✓ Learn on how to treat water from households with educational literate and females

## Abbreviations

AOR: Adjusted odds ratio
COR: Crude odds ratio
HH: Household
MDG: Millennium Development Goal
WHO: Word Health Organization
